# Analysis of pre-service teachers’ knowledge about the entrepreneurial competence: a case study of a Spanish university

**DOI:** 10.3389/fpsyg.2023.1279705

**Published:** 2023-12-05

**Authors:** Arantza Arruti, Estibaliz Benitez, Jessica Paños-Castro

**Affiliations:** ^1^Department of Education, Faculty of Education and Sports, University of Deusto, Bilbao, Spain; ^2^La Inmaculada MSJO, Bilbao, Spain

**Keywords:** entrepreneurship education, EntreComp, EntreCompEdu, entrepreneur teacher, Spain, higher education, university

## Abstract

**Introduction:**

This research is based on the role played by the entrepreneurial competence (EC), entrepreneurial education (EE) and teachers in the social, economic and cultural development of a society. The general objective of the study is to analyze the level of knowledge pre-service, those who are studying, or have recently studied, the Bachelor’s Degree in Primary Education at the University of Deusto (Spain) (DPEUD) have about the EC.

**Methods:**

A questionnaire, based on EntreComp Framework, underwent expert validation and was applied to a sample of 304 students.

**Results:**

The data showed that 25% of the respondents believed that EE was related to educating through entrepreneurship; more than 45% did not know about EntreComp and EntreCompEdu, whereas only three participants were aware of how to use them; and more than 10% of the pre-service teachers did not consider assessing the CE.

**Discussion:**

These results lead to the conclusion that there is a need for EE to form part of the different national teacher training strategies; and for policy makers to include EE in the different educational frameworks, laws and decrees. In addition, it can be concluded that social, cultural and economic value can be created through entrepreneurial actions; that EC should be assessed; and that teachers should motivate students to share and implement entrepreneurial ideas and actions.

## 1 Introduction

Several laws, decrees, orders and guidelines have been implemented in Spain in recent years which have had an impact on the development of initiative and entrepreneurship, on the provision of high-quality entrepreneurial education (EE), and on the creation of an entrepreneurial culture. Some examples are Organic Law 10/2002 (Jefatura del Estado, 2002), the first legal instrument to address entrepreneurship, Organic Law 2/2006 (Jefatura del Estado, 2006), Organic Law 8/2013 (Jefatura del Estado, 2013), Organic Law 3/2020 (hereinafter, Law 3/2020) (Jefatura del Estado, 2020) and the most recent Royal Decree 157/2022 of 1 March, establishing the organisation and basic curriculum contents of Primary Education (hereinafter, Decree 157/2022) (Ministerio de Educación y Formación Profesional, 2022). All of them included ‘a sense of initiative and entrepreneurship’ and/or the entrepreneurial competence (EC). While Giménez Beut (2022) stated that there are different approaches to the definition of the CE, Law 3/2020 (Jefatura del Estado, 2020) emphasized the need to take into account both the particular situation and needs in order to turn them into opportunities, with a view to promoting ethical commitment, sustainable development and active citizenship.

The need to include entrepreneurship in laws and educational institutions stems from the need to train entrepreneurial professionals who are able to work in teams and to manage uncertainty, take risks, collaborate, and participate in projects within a framework of values that promotes sustainability and citizenship, among other things (GAZE, 2011).

EE seeks to foster students’ personal and social development in order to help them develop competences such as teamwork, enhance their creativity, learn to put their main qualities into practice, have an active attitude, and show resilience in the face of adversity, among other things (Grigg, 2020). The aim of EE is no longer only to provide students with training on how to set up a business, but to promote an entrepreneurial mindset among and so prepare them for the complex socio-economic ecosystem that humanity is facing (Paños, 2017).

The European Commission (2010) analyzed different strategies and action plans to identify good practices in its efforts to ensure high standards for EE, and proposed a consistent model of progression with indicators to serve as a benchmark at the European level. Its conclusions pointed to the need to establish a clear definition of EE; to show strong inter-ministerial cooperation in the articulation of strategies; and to consult with key stakeholders in order to achieve the highest possible degree of understanding and dissemination. In addition, it is recommended that entrepreneurship be included as a key competence in all curricular areas and that funds be made available to develop teacher training strategies.

The research question is as follows: what is the level of knowledge pre-service teachers, those who are studying or have recently studied, the Bachelor’s Degree in Primary Education at the University of Deusto (Spain) (DPEUD) have about the EC? This question is specified in the objectives and hypotheses listed in section 3.1. Objectives and Hypotheses.

The rest of the study is organized as follows. The following section presents the literature review. It consists of four subsections (The entrepreneurial competence and entrepreneurial education; Teacher training in the entrepreneurial competence; The EC in initial teacher education and Teacher training in the entrepreneurial competence). After it, the method is presented and the empirical results and discussion are suggested. The study ends with the main conclusions.

## 2 Literature review

### 2.1 The entrepreneurial competence and entrepreneurial education

Entrepreneurship is a significant area of knowledge, learning and research, and is considered to be a key element for economic, social and sustainable development and a fundamental instrument for the individual’s overall education (United Nations, 2015). Since the publication of the Entrepreneurship 2020 Action Plan (Comisión Europea, 2013), the EC has been considered to be one of the pillars of youth education. It is deemed to ensure successful outcomes in a changing, inclusive and critical society (Sanahuja et al., 2020), and is therefore included in the curricula worldwide (González-Tejerina and Vieira, 2021). When the European Parliament and Council of the European Union (2006) established the eight key competences for lifelong learning, the EC was included among them. This was defined as acting upon opportunities and ideas and transforming them into financial, cultural or social value for others (Vestergaard et al., 2012).

As Kuratko and Hodgetts (2004) and Deveci and Seikkula-Leino (2018) argued, the concept of entrepreneurship is currently not only linked to the process of job creation, but also takes into account how this affects individual behavior in different job-related activities.

As is the case with entrepreneurship, EE is a constantly evolving concept (Bueckmann Diegoli et al., 2017). Moreover, the way it is approached continues to cause confusion among those involved in its design and development (Lackéus, 2015).

The study carried out by Paños-Castro and Arruti (2019) on the EC and the entrepreneurial behavior of university teachers of primary education (PE) showed that the EC is not sufficiently integrated into the curricula, and that university lecturers do not hold it to be one of the key competences for future PE teachers. Furthermore, several studies have shown that there is very little EC content in the curricula of PE degrees in Spain. The study carried out by Arruti et al. (2021) analyzed 631 competences of these degrees at Jesuit universities in Spain and concluded that entrepreneurship was only within 30% of the competences. Cárdenas Gutiérrez and Azqueta Díaz de Alda (2022) noted that in this degree there was no specific subjects on entrepreneurship, it was only found in the specialized pathways with a clear business focus in the Master’s Degree for teachers. Paños-Castro et al. (2022) also examined 68 curricula of PE degrees in Spanish universities, including a total of 6,262 competences and 655 methodologies, and concluded that the EC appeared in 33.82% of the total (909 competences).

What seems to be clear is that university lecturers should provide students with the EC (Miço and Cungu, 2023) and allow them ‘to search (*sic*) the ideal content of entrepreneurship education as well as the manner in which it should be handled’ (Deveci and Seikkula-Leino, 2018, p. 132). According to Wach (2014), education can ultimately have a positive impact on entrepreneurship. In this regard, Deveci and Seikkula-Leino (2018, p. 133) emphasized that EE ‘must be included in curricula based on a fundamental philosophy, but it should not be treated as just an ordinary topic covered in curricula’, and Hamid (2013) underlined that different research has advocated that it must be handled through a cross-curricular approach.

### 2.2 Teacher training in the entrepreneurial competence

While there is not much research on EE and the training of future teachers (Arruti and Paños-Castro, 2019), Cárdenas Gutiérrez and Azqueta Díaz de Alda (2022) stated that it can be affirmed that their training is a key element for the development of entrepreneurial education, as there is a marked shortage of training for pre-service teachers regarding the EC. For Deveci and Seikkula-Leino (2018), ‘it is considered highly important that pre-service teachers become acquainted with entrepreneurship education during pre-service training’ (p. 137). However, according to González-Tejerina and Vieira (2021), the implementation of EC entails a series of difficulties, including lack of teacher training, lack of resources, ineffective methods, unsuitable design, poor planning and lack of assessment.

Along these lines, Cárdenas Gutiérrez and Azqueta Díaz de Alda (2022) approached EE from a humanist model that sought to help people to think, reflect and contribute to personal improvement; to promote citizen values; to develop initiative and the drive to take actions that are difficult and require willingness to accept responsibility and step up to new challenges; and to be open to real-life situations, which triggers the need for people to become increasingly interested in circumstances outside themselves in order to contribute to the improvement of their immediate environment.

Furthermore, different authors have stressed the need for good EC program design, transforming curricula, incorporating clear objectives, and modifying teaching-learning methodologies and the assessment system (Maritz and Brown, 2013; Kassean et al., 2015; European Commission, 2016). In order to promote a shared vision of the EC, the European Commission developed the EntreComp framework (Bacigalupo et al., 2016), which seeks to provide every citizen with transversal skills and key competences that bring new opportunities for personal fulfilment and development, social inclusion, active citizenship and employment. The model consists of three competence areas (Ideas and Opportunities, Resources, and Into Action), 15 competences, an eight-level progression model and a comprehensive list of 442 learning outcomes (Bacigalupo et al., 2016; McCallum et al., 2018).

Based on the European EntreComp framework, Grigg (2020) published the EntreCompEdu model, which aims to enhance the professional development of primary, secondary and vocational education teachers through a skills framework that enables them to understand and improve their ECs in relation to EE, and to help them shape a creative and entrepreneurial mindset. EntreCompEdu consists of five areas of competence (entrepreneurial knowledge and understanding, planning and organisation, teaching and training, assessment, and professional learning) and 17 competences. It is based on six principles according to which knowledge is socially constructed through interaction and experience, and acquired through an iterative process (Grigg, 2020).

Stemming from the conviction that the challenges of the 21st century also require a coherent response in relation to teacher training, Cárdenas Gutiérrez and Azqueta Díaz de Alda (2022) stated that without this teacher training, it will be difficult for pupils in the lower educational stages to acquire the key competences for personal, social and professional development to live up to current and future situations.

This may be the reasoning behind the model of EC for teachers called ‘EntrepComEdu’ (a holistic model of teacher entrepreneurial competence) proposed by Sanz Ponce and Núñez Canal (2022), based on Caena and Redecker's (2019) model of digital competence. The model is divided into three blocks: professional competences, pedagogical competences, and impact on students’ competence.

### 2.3 The EC in initial teacher education

Arruti et al. (2021) conducted a study on the extent to which the EntreComp and EntreCompEdu competences were reflected in the competences to be developed by primary education degree students at the different Jesuit universities in Spain, which produced the following results, among others: the EntreComp competence areas were included to a greater extent in the different degree competences; and participants were not aware of the first and fourth EntreCompEdu competence areas, i.e., knowledge and entrepreneurial understanding, and assessment. This means that a number of competences were not included among those to be developed, notably including: knowing about entrepreneurial education, valuing entrepreneurial education for all, understanding how learners acquire entrepreneurial competences, checking and reporting on progress, sharing feedback, and recognizing progress and achievement.

Based on the above considerations, this study analyses pre-service (primary education) teachers’ perceived level of knowledge in relation to the areas of Entrepreneurial Knowledge and Understanding, and Assessment of the EntreCompEdu framework.

#### 2.3.1 Entrepreneurial knowledge and understanding, and assessment

On the one hand, according to EntreCompEdu, Entrepreneurial Knowledge and Understanding is the area that focuses on understanding EE, valuing the opportunities it can offer to learners, and understanding how teachers help learners to acquire EE. As can be seen in Table 1, this is composed of three sub-competences (Grigg, 2020) which, in turn, correspond to EntreComp area 1, spotting opportunities, creativity, vision (Bacigalupo et al., 2016).

**Table 1:** Entrepreneurial knowledge and understanding, and assessment according to EntreCompEdu and its correspondence with EntreComp.

**EntreCompEdu**		**EntreComp**		
1. PROFESSIONAL KNOWLEDGE AND UNDERSTANDING OF ENTREPRENEURIAL EDUCATION		1. IDEAS AND OPPORTUNITIES		
1.1 Knowing entrepreneurial education	Continually developing my knowledge of entrepreneurial education, including the EntreComp framework.	1.1. Spotting opportunities	Use your imagination and abilities to identify opportunities for creating value	Identify and seize opportunities to create value by exploring the social, cultural and economic landscape Identify needs and challenges that need to be met Establish new connections and bring together scattered elements of the landscape to create opportunities to create value
1.2 Valuing entrepreneurial education	Understanding the relevance and potential of entrepreneurial education in my teaching and students’ learning.	1.2. Creativity	Develop creative and purposeful ideas	Develop several ideas and opportunities to create value, including better solutions to existing and new challenges Explore and experiment with innovative approaches Combine knowledge and resources to achieve valuable effects
1.3 Understanding how learners develop entrepreneurial competences	Identifying my students’ needs, interests and starting-points and using this knowledge to inform how I approach entrepreneurial education.	1.3. Vision	Work towards your vision of the future	Imagine the future Develop a vision to turn ideas into action Visualize future scenarios to help guide effort and action
4. ASSESSMENT		3. INTO ACTION		
4.1 Checking and reporting on progress	Monitoring and reporting on what students know, understand and can do in their entrepreneurial learning.	3.2. Planning and management	Prioritize, organize and follow up	Set long-, medium- and short-term goals Define priorities and action plans Adapt to unforeseen changes
4.2 Sharing feedback	Ensuring that students know what and how they need to improve their entrepreneurial learning and are becoming increasingly involved and engaged in assessing their own progress.	3.3. Coping with uncertainty, ambiguity and risk	Make decisions dealing with uncertainty, ambiguity and risk	Make decisions when the result of that decision is uncertain, when the information available is partial or ambiguous, or when there is a risk of unintended outcomes Within the value-creating process, include structured ways of testing ideas and prototypes from the early stages, to reduce risks of failing Handle fast-moving situations promptly and flexibly
4.3 Recognizing progress and achievement	Providing opportunities for students to share the evidence of their entrepreneurial learning journey through two-way communication with a range of audiences in and beyond the school.	3.4. Working with others	Team up, collaborate and network	Work together and co-operate with others to develop ideas and turn them into action Network Solve conflicts and face up to competition positively when necessary
		3.5. Learning through experience	Learn by doing	Use any initiative for value creation as a learning opportunity Learn with others, including peers and mentors Reflect and learn from both success and failure (your own and other people’s)

Developed by the authors, based on EntreComp (Bacigalupo et al., 2016; Grigg, 2020).

On the other, according to EntreCompEdu, Assessment is the competence that focuses on explaining the importance of involving learners in their own assessment and development in order to have the opportunity to be aware of their failures and strengths, and to have the necessary tools to be able to improve their weaknesses. Table 1 shows that this is composed of three sub-competences (Grigg, 2020), which correspond to EntreComp area 3: planning and management, coping with uncertainty, ambiguity and risk, working with others and learning through experience (Bacigalupo et al., 2016).

## 3 Method

### 3.1 Objectives and hypotheses

The general objective of the study is to analyze the level of knowledge pre-service teachers (those who are studying or have recently studied, the Bachelor’s Degree in Primary Education at the University of Deusto (Spain) (DPEUD) have about the EC. These included students in years 1 to 4 of the 2021/2022 cohort and those cohorts who completed their degree in the two academic years before (hereinafter, students of one cohort/year or another).

This study had two specific objectives:

To analyze the level of knowledge about Entrepreneurial Knowledge and Understanding (EKU) (a sub-competence of the EC) as perceived by students who are taking or have recently taken the DPEUD, paying special attention to how EE is defined, the European EC benchmark frameworks Entrecomp and EntreCompEdu, and the social, cultural and economic value of entrepreneurial actions in schools.

To analyze the level of knowledge about Assessment (a sub-competence of the EC) as perceived by students who were taking or have recently taken the DPEUD, paying special attention to the consideration of students’ needs, interests and talents in the development of the EC, the need to assess and give feedback about the EC, and the need to identify opportunities and groups to share and implement entrepreneurial ideas and actions.

There were 6 hypotheses:

*H1*: The level of self-perceived knowledge of EKU in relation to EE is not associated with students' belonging to one cohort/academic year or another.

*H2*: The level of self-perceived knowledge about EKC in relation to the European EC benchmark frameworks Entrecomp and EntreCompEdu is not associated with students' belonging to one cohort/academic year or another.

*H3*: The level of self-perceived knowledge about EKC in relation to the social, cultural and economic value of entrepreneurial actions in schools is not associated with students' belonging to one promotion/academic year or another.

*H4*: The self-perceived level of knowledge about Assessment in relation to students' needs, interests and talents in the acquisition of the EC is not associated with students' belonging to one cohort/academic year or another.

*H5*: The self-perceived level of knowledge about Assessment in relation to the need to assess and give feedback on the EC is not associated with students' belonging to one cohort/academic year or another.

*H6*: The self-perceived level of knowledge about Assessment in relation to the need to identify opportunities and groups to share and implement entrepreneurial ideas and actions is not associated with students' belonging to one cohort/academic year or another.

### 3.2 Instruments

A literature review was conducted, followed by the designing of a questionnaire based on the EntreComp and EntreCompEdu frameworks (Bacigalupo et al., 2016; McCallum et al., 2018; Grigg, 2020). The areas to be included in the questionnaire were selected at that stage, namely, entrepreneurial knowledge and understanding, and assessment (Arruti et al., 2021). Subsequently, the questionnaire was validated according to the guidelines laid out by Ding and Hershberger (2002). The validation process was carried out by eight experts from the field of education and entrepreneurship, who were asked to indicate the suitability, relevance and clarity of the items, as well as the need to add any other item to the questionnaire.

After the experts’ views were gathered, the percentage of agreement was analyzed by averaging across the three categories. Items were only included in the questionnaire if between 50 and 70% of the expert panel had given them the highest score. After the process, no items had to be removed, as they were all positively rated. Only a few grammatical corrections to some items were necessary to improve clarity of understanding. It was also decided to add an item that was identified as important by 4 of the 8 experts.

After the expert panel completed their analysis, the questionnaire consisted of three blocks: two initial questions on general personal data of participants (academic year or cohort to which they belonged and gender); questions 1–6 on Entrepreneurial Knowledge and Understanding; questions 7–10 on Assessment. Questions 1–10 were on a Likert-type scale of 1–4. Participants were required to answer all questions. They could also write any comments they wished at the end of the questionnaire.

Finally, a pilot study was conducted to examine the overall performance of the questionnaire. In this study, five potential participants from the PE Degree participated and corroborated the degree of understanding of the instrument’s items.

### 3.3 Sample and data collection

The population under study was university students who were completing their PE degree (years 1–4) in academic year 2021/2022), as well as those from the classes that completed their studies in the 2019/2020 and 2020/2021 academic years: 45 students from the 2019/2020 cohort; 47 from the 2020/2021 cohort. There were 46 participants from the 1st year; 58 from the 2nd year; 59 from the 3rd year; and 49 from the 4th year in the 2021/2022 academic year, which yielded a total of 304 potential participants.

The questionnaire was sent out via Google Forms on 9 May 2022 and, after a reminder, it closed on 31 May 2022. Before the questionnaire was sent, students were informed about the study in two ways: students in the 2021/2022 academic year were informed by e-mail and, when possible, verbally in the last 5 min of a classroom session; and students in the 2019/2020 and 2020/2021 groups were sent a brief message via WhatsApp using the snowball methodology. Ethical research principles were followed, and anonymity and voluntary participation were respected at all times.

The final sample consisted of 182 students, 59.8% of the total sample. Table 1 shows the participation rates with respect to the general data. The highest participation rate was found in the 3rd year group of the 2021/2022 academic year and the lowest was found among the 2019/2020 cohort. The percentage of female participants was much higher than that of male participants, not surprisingly, as the percentage of female students in the PE degree was much higher than that of male students (in the 2021/2022 academic year, 80% of students in the 2021/2022 promotion were female and 20% were male, a proportion that remained in every year from 1st to 4th) (Table 2).

**Table 2:** Participant sample data – Count (% within gender).

		Gender			Total
		Female	Male	Others	
Cohort/Academic year	1	18 (12.2%)	3 (9.1%)	0 (0.0%)	21 (11.5%)
	2	11 (7.4%)	2 (6.1%)	0 (0.0%)	13 (7.1%)
	3	18 (12.2%)	7 (21.2%)	0 (0.0%)	25 (13.7%)
	4	41 (27.7%)	7 (21.2%)	1 (100%)	49 (26.9%)
	5	26 (17.6%)	11 (33.3%)	0 (0.0%)	37 (20.3%)
	6	34 (23.0%)	3 (9.1%)	0 (0.0%)	37 (20.3%)
Total		148 (100%)	33 (100%)	1 (100%)	182 (100%)

Cohort/Academic year: 1: 2020/2021 cohort; 2: 2019/2020 cohort; 3: Year 12,021/2022 cohort; Year 22,021/2022 cohort; Year 32,021/2022 cohort; Year 42,021/2022 cohort.

Different tests were conducted to check whether the sample obtained was truly representative and draw some conclusions. The sample was considered to be representative if confidence level was 95% and the margin of error was 5%. According to Sierra Bravo (1989), the sample size has to comply with four factors: total sample size, confidence level, estimation error and standard deviation. The total number of potential participants in this study was 304; therefore, for a confidence level of 95% and a margin of error of 5%, the required sample size was 171. As the final sample had, 182 (59.2%) participants, it was representative.

Data were analyzed using the SPSS statistical package. The questionnaire was subjected to an internal consistency analysis using Cronbach’s alpha. Nunnally (1978) recommended a minimum level of 0.7 to be considered good. The total Cronbach’s alpha coefficient for the questionnaire was 0.739, which indicates adequate internal consistency and reliability.

## 4 Empirical results and discussion

With regard to statistical data, Table 3 shows the mean, deviation and variance for the 10 questions. The standard deviation was greater than 1 in 3 in questions (questions 5, 7, and 8). It was shown that the lowest averages corresponded to questions 2, 3, and 4, which referred to the knowledge and application of the EntreComp and EntreCompEdu EC frameworks, the EC sub-competences and the purpose and benefits of EE.

**Table 3:** Statistical data for the 10 questions.

		**P1**	**P2**	**P3**	**P4**	**P5**	**P6**	**P7**	**P8**	**P9**	**P10**
***N***	**Valid**	182	182	182	182	182	182	182	182	182	182
	**Lost**	0	0	0	0	0	0	0	0	0	0
**Average**		3.45	1.22	1.16	1.44	2.55	2.91	2.95	2.73	3.16	3.12
**Median**		4	1	1	1	2	3	3	3	3	3
**Mode**		4	1	1	1	2	3	4	2	4	4
**Std. deviation**		0.825	0.591	0.495	0.685	1.154	0.823	1.223	1.029	0.943	0.93
**Variance**		0.68	0.349	0.245	0.469	1.331	0.677	1.495	1.06	0.89	0.865

Standard error of skewness: 180; Standard error of kurtosis: 0.358.

Table 4 shows the result of the Pearson’s chi-square test, which indicates that, with respect to cohort/academic year, chi-square was greater than 0.05 in questions 1, 5, 6, 7, 9, and 10, and in questions 2, 3, 4, and 8 it was lower than 0.05. These data help to test whether variables were associated or not; in the case of this study whether there was an association between the perceived level of knowledge of the ECs and membership of one cohort/academic year or another.

**Table 4:** Pearson’s chi-square test results according to cohort/academic year.

	**Q1**	**Q2**	**Q3**	**Q4**	**Q5**	**Q6**	**Q7**	**Q8**	**Q9**	**Q10**
**Value**	15.779	32.46	25.816	58.283	20.362	12.998	14.885	26.573	8.589	16.622
**df**	15	15	15	15	15	15	15	15	15	15
**Asymptotic significance (two-tailed)**	0.397	0.006	0.04	<0.001	0.158	0.602	0.46	0.032	0.898	0.342

Number of valid cases: 182.

Regarding the overall scores obtained in each question (questions 1 to 10), Table 5 shows that between 62.1 and 89% believed that EE was mainly related to educating to develop ECs; but that they did not know the two, or one of the two, European EC benchmark frameworks (Entrecomp and EntreCompEdu); they did not know all the sub-competences that make up the EC; and they did not know exactly what EE involves.

**Table 5:** Total data and percentages by question (1–10).

Option	Q1	Q2	Q3	Q4	Q5	Q6	Q7	Q8
1	8	155	162	118	38	9	41	23
	4.40%	85.20%	89.00%	64.80%	20.90%	4.90%	22.50%	12.60%
2	15	17	12	52	65	43	17	58
	8.20%	9.30%	6.60%	28.60%	35.70%	23.60%	9.30%	31.90%
3	46	7	7	8	19	85	34	46
	25.30%	3.80%	3.80%	4.40%	10.40%	46.70%	18.70%	25.30%
4	113	3	1	4	60	45	80	55
	62.10%	1.60%	0.50%	2.20%	33.00%	24.70%	49.50%	30.20%

Between 31.9 and 35.7% recognized that, they did not feel able to assess the EC even though it is important to explicitly do so. They supported or said that they would support entrepreneurial actions of social, cultural or economic value that they may be able to carry out when they teach in a school.

Around 50% of the participants, specifically, between 46.7 and 49.5%, believed that they should have to develop the EC in PE; that they encouraged or would encourage their pupils to identify possible groups of people with whom to share and implement entrepreneurial ideas and actions; that during the assessment process, they helped or would help students pupils to self-evaluate, i.e., they manage their learning, effort, achievements and progress, and reflect critically on their entrepreneurial learning process; and finally, they believed that EE involves taking the needs, interests and talents of pupils as a starting point in order to work alongside them and guide in the acquisition of the ECs in an autonomous way.

Based on the research question posed before, the results achieved for each question are shown below. These are related to the hypotheses and specific objectives, as follows: Questions 1–6 with hypotheses 1–3 and, consequently, with specific objective 1 (knowledge about Entrepreneurial Knowledge and Understanding); Questions 7–10 with hypotheses 4–6 and, consequently, with specific objective 2 (knowledge about Assessment).

The results are analyzed by question below. Question 1 (chi-square 0.397) asked whether EE was believed to be related to: educating about entrepreneurship; educating for entrepreneurship; educating through entrepreneurship; or educating to acquire ECs. As can be seen in Table 6, more than half of the participants considered that EE was mostly related to educating to develop ECs, whereas more than 25% considered that it was related to educating through entrepreneurship.

**Table 6:** Results.

**Q**			**Cohort/Academic year**						**T**	**Q**	**Cohort/Academic year**						**T**
			1	2	3	4	5	6									
1	1	C	0	0	2	3	2	1	8	7	4	2	7	9	8	11	41
		%	0	0	8	6.1	5.4	2.7	4.4		19	15.4	28	18.4	21.6	29.7	22.5
	2	C	1	0	1	3	4	6	15		0	0	1	5	7	4	17
		%	4.8	0	4	6.1	10.8	16.2	8.2		0	0	4	10.2	18.9	10.8	9.3
	3	C	5	5	6	11	14	5	46		4	1	6	11	6	6	34
		%	23.8	38.5	24	22.4	37.8	13.5	25.3		19	7.7	24	22.4	16.2	16.2	18.7
	4	C	15	8	16	32	17	25	113		13	10	11	24	16	16	90
		%	71.4	61.5	64	65.3	45.9	67.6	62.1		61.9	76.9	44	49	43.2	43.2	49.5
3	1	C	16	10	25	46	32	33	162	8	2	0	8	5	3	5	23
		%	76.2	76.9	100	93.9	86.5	89.2	89		9.5	0	32	10.2	8.1	13.5	12.6
	2	C	3	3	0	2	1	3	12		7	4	5	16	15	11	58
		%	14.3	23.1	0	4.1	2.7	8.1	6.6		33.3	30.8	20	32.7	40.5	29.7	31.9
	3	C	1	0	0	1	4	1	7		2	3	6	11	8	16	46
		%	4.8	0	0	2	10.8	2.7	3.8		9.5	23.1	24	22.4	21.6	43.2	25.3
	4	C	1	0	0	0	0	0	1		10	6	6	17	11	5	55
		%	4.8	0	0	0	0	0	0.5		47.6	46.2	24	34.7	29.7	13.5	30.2
4	1	C	12	2	24	36	23	21	118	9	1	1	1	2	3	2	10
		%	57.1	15.4	96	73.5	62.2	56.8	64.8		4.8	7.7	4	4.1	8.1	5.4	5.5
	2	C	4	7	1	13	12	15	52		2	4	3	13	8	8	38
		%	19	53.8	4	26.5	32.4	40.5	28.0		9.5	30.8	12	26.5	21.6	21.6	20.9
	3	C	3	4	0	0	1	0	8		5	3	10	10	8	10	46
		%	14.3	30.8	0	0	2.7	0	4		23.8	23.1	40	20.4	21.6	27	25.3
	4	C	2	0	0	0	1	1	4		13	5	11	24	18	17	88
		%	9.5	0	0	0	2.7	2.7	2.2		61.9	38.5	44	49	48.6	45.9	48.4
5	1	C	5	1	7	12	7	6	38	10	1	0	0	1	0	2	4
		%	23.8	7.7	28	24.5	18.9	16.2	20.9		4.8	0	0	2	0	5.4	2.2
	2	C	4	3	8	15	17	18	65		3	5	6	15	17	11	57
		%	19.0	23.1	32	30.6	45.9	48.6	35.7		14.3	38.5	24	30.6	45.9	29.7	31.3
	3	C	1	2	0	8	5	3	19		3	4	5	8	9	6	35
		%	4.8	15.4	0	16.3	13.5	8.1	10.4		14.3	30.8	20	16.3	24.3	16.2	19.2
	4	C	11	7	10	14	8	10	60		14	4	14	25	11	18	86
		%	52.4	53.8	40	28.6	21.6	27	33.0		66.7	30.8	56	51	29.7	48.6	47.3
6	1	C	1	0	2	1	3	2	9								
		%	4.8	0	8	2	8.1	5.4	4.9								
	2	C	3	2	10	11	10	7	43								
		%	14.3	15.4	40	22.4	27	18.9	23.6								
	3	C	11	6	11	26	14	17	85								
		%	52.4	46.2	44	53.1	37.8	45.9	46.7								
	4	C	6	5	2	11	10	11	45								
		%	28.6	38.5	8	22.4	27	29.7	24.7								

Questions (Q) 1 and 3–10. C, count; %, % within cohort/academic year; T, total.

In relation to Question 2 on whether or not the respondent was aware of the European EC benchmark frameworks Entrecomp and EntreCompEdu, and whether they knew how to use them, the results are shown in Table 7. A 45.6% of the participants are not aware either of the European frameworks, compared to 8.2% who are aware of both, and only 1.6% know how to use them.

**Table 7:** Results.

Participants were aware of			Participants were not aware of	Participants knew how to use		
EntreComp	EntreCompEdu	Both	Neither	EntreComp	EntreCompEdu	Both
41	33: All but 1 F (preferred not to answer).	15	83: 22 (M): 1: 2; 2: 1; 3: 7; 4: 3; 5: 6; 6: 3 61 (F); 1: 7; 2: 5; 3: 10; 4: 23; 5: 5; 6: 11	3. 2 were in their 1st year (F), 1 was in his 3rd year (M).	4: 3 were in their 3rd year (two M and one F), and 1 was in her 4th year (F).	3: 1 was in her 2nd year and two were in their 4th year (F).
22.5%	18.1%	8.2%	45.6%	1.6%	2.1%	1.6%

Question 2. F, female; M, male.

Question 3 (chi-square 0.40) was related to question 2 and referred to knowledge about the sub-competences that make up the EC and whether they knew how to incorporate them into their work. As can be seen in Table 6, the most striking figure was that of the 1st year students in the 2021/2022 academic year, who stated that they neither knew nor knew how to integrate the two European EC benchwork frameworks.

Question 4 (chi-square < 0.001) was about whether they knew what EE is, its purpose, benefits and how to contribute to it as a teacher. In this case, as can be seen in Table 6, more than 60% stated that they did not know exactly what EE involves. In this group, the students in the 2019/2020 cohort were in the minority and were also the group that was most aware of the purpose of the EE and the benefits it can bring to their pupils, with the highest percentage (more than 50%). In fact, the first-year group of the 2021/2022 cohort was the least aware of the purpose and benefits of EE.

Regarding question 5 (chi-square 0.158), concerning the social, cultural or economic value of entrepreneurial actions in schools, Table 6 shows that only the 2020/2021 and 2019/2020 academic year groups stated that they not only knew the value of entrepreneurial actions in schools, but also that they encouraged or would encourage their students to use this type of action (with more than 50% each). Also noteworthy were the groups of 3rd and 4th year students in the 2021/2022 cohort, as almost 50% of each group stated that they would support this type of entrepreneurial action.

In the case of question 6 (chi-square 0.602), the different groups believed that the ECs (or at least some of them) should be learnt in PE, as can be seen in Table 6. Still 4.9% believed that not much can be done to contribute to the development of pupils’ ECs in PE. Almost 25% reported that they would work on the ECs with their pupils.

In relation to question 7 (chi-square 0.460) on the need to be aware of the needs, interests and talents of pupils in order for them to acquire ECs, almost 50% of the participants agreed that the EC involves taking the needs, interests and talents of pupils as a starting point in order to guide them and work with them to help them autonomously acquire ECs (see Table 6). The lowest percentage was found among participants who believed that EE involves taking the needs, interests and talents of pupils in order to plan EC acquisition.

Question 8 (chi-square 0.032) asked about assessing the ECs. More than 10% of the participants did not consider assessing the ECs, compared to 31% who believed that it important to explicitly assess the ECs, but did not consider themselves to be able to do so, while more than 35% already did assess or would assess the ECs (Table 6).

With regard to Question 9 (chi-square 0.898), on whether or not teachers should assess the ECs, Table 6 shows that 5.5% believed that PE teachers should not assess the ECs, compared to almost 50% who were in favor of helping pupils during the assessment process so that they could self-assess, manage their learning, effort, achievements and progress, and reflect critically on their entrepreneurial learning process. The rest of the participants also considered it necessary to assess the process of EC acquisition.

Finally, in Question 10 (chi-square 0.342), concerning opportunities to share and implement entrepreneurial ideas and actions, almost 50% said that they encouraged or would encourage their pupils in the future to identify possible groups of people with whom to share and implement entrepreneurial ideas and actions, while more than 30% of the respondents believed that it is important to provide pupils with these opportunities (Table 6).

## 5 Discussion

In relation to Question 1, based on the results, H1 on how EE is understood by the participant pre-service teachers can be accepted; it does not seem that belonging to one cohort/academic year or another was associated with the level of self-perceived knowledge of the EKU in relation to EE. It was striking that more than 60% of respondents stated that EE is related to educating to acquire the ECs as opposed to other options, although still more than 25% of the participants considered that EE is related to educating through entrepreneurship. In this respect, one of the 4th year students of the 2021/2022 cohort noted the following question in the comments section: ‘Do you think that there are teachers who still believe that entrepreneurship is only based on the business sphere?’

According to Guillén Tortajada and Jimenez Martínez (2022), a transformation of the education system is needed to train education professionals in the ECs, whereby education programs will include this kind of education from the outset (Correia et al., 2010; Konokman and Yelken, 2014). In fact, according to the European Training Foundation (2010), EE is not yet part of all national teacher training strategies, although Žibėnienė (2012), among others, recognized that it is included in many national curricula, and the European Union promotes the inclusion of the EC in educational curricula (González-Tejerina and Vieira, 2021). In the case of Spain, under Royal Decree 157/2022 (Ministerio de Educación y Formación Profesional, 2022) the EC is listed as one of the key competences to be acquired by pupils from primary education onwards.

Regarding this question, it is worth noting the comment that one of the students of the 2019/2020 cohort wrote in the comments section:

‘I think we teachers know less about entrepreneurship than we should because we were not taught it at school. When I assess myself, I recognize that I know little about the subject, but I am willing to learn whatever is necessary to ensure that the children I teach will learn concepts and knowledge of important life issues such as this one.'

Focusing on Question 4, H1 must be rejected. In fact, in the case of this research, the older the students, the more aware they were of the purpose and benefits of the EC. This may be due, among other reasons, to the fact that during the academic years 2019/2020 and 2020/2021, between 10 and 15 students participated in EC awareness and implementation programs. There is still a long way to go for future teachers to know not only what the purpose and benefits of EE are, but also how to contribute to EE within their professional practice.

Several students from the 1st to the 3rd years of the 2021/2022 cohort emphasized in the comments section that it was important that they be taught about entrepreneurship, because in some cases ‘it does not even ring a bell’ or ‘because I really have no knowledge of this (...) and I think it could be interesting and important for future teachers to apply it in primary education’, and even that ‘there is no point in Entrepreneurial Education if they do not help you in any way to make it happen in the future’.

As Deveci and Seikkula-Leino (2018) stated, there are many studies that advocate including EE in the teacher training curriculum (Chikodi Ebo et al., 2023), and therefore, starting the training in the degree program will enable them to become teachers. In fact, different studies have shown that teachers do not feel sufficiently supported and prepared to implement EE (Mattila et al., 2009; Seikkula-Leino et al., 2010; Ruskovaara and Pihkala, 2013; Deveci and Seikkula-Leino, 2018; Miço and Cungu, 2023). In fact. Grigg (2020) found that 27% of participants in the EntreCompEdu project claimed to have no prior experience of EE; Ruskovaara and Pihkala (2013) found that 196 teachers out of the 521 who took part in the project had not received any training in EE; and Miço and Cungu (2023) highlighted that only 44.6% reported having received training in the EC that enabled them to include it in the subject they teach, and 71% stated that they had not received any training in EE during their studies at university. In addition, only 29.6% of the participants considered themselves sufficiently competent in the EC.

In a recent study carried out by Arruti and Paños-Castro (2023) involving 326 teachers from different non-university educational levels, more than half of the respondents indicated that they had no knowledge of EE but could imagine what it meant. As Penaluna and Penaluna (2015, p. 7) stated ‘there are currently no definitive pedagogical guidelines for entrepreneurship education within schools’. It is necessary to include EE training programs in all educational levels.

The results obtained in relation to Question 2 concerning the EC Entrecomp and EntreCompEdu benchmark frameworks indicate that H2 must be rejected, that is, it seems that belonging to one cohort/academic year or another was related to the level of self-perceived knowledge of EKU in relation to the Entrecomp and EntreCompEdu EC benchmark frameworks. More than 45% of students knew neither of the two frameworks and only 3 people knew how to use both, whereas only 15 people knew both frameworks. These data only confirm that there is still a long way to go. In fact, students in the first year in the 2021/2022 academic year reported that they neither knew of, nor knew how to integrate, the two benchmark frameworks. These data are different from those obtained by Grigg (2020), who found that of the 308 teachers participating in the EntreCompEdu pilot project, 32% had never heard of EntreComp. The same was true for the research conducted by Seikkula-Leino et al. (2021), who found that, of the 348 professionals (policy makers, educators, and other actors) from 47 countries who participated in their study, almost half of the respondents, 49.1%, were aware of the EntreComp framework, 21.3% had heard of it, and 29.3% had not heard of EntreComp at all. In the case of Spain (a total of 36 people participated), 63.9% said they were aware of EntreComp. These data also contrast with those found by Arruti and Paños-Castro (2023), who in their study found that more than 70% of the 326 in-service teachers who participated in their study said that they had never heard of the EntreComp and EntreCompEdu benchmark frameworks.

In terms of Questions 5 and 6, the results show that H3 must be accepted, although the dispersion of data was very high in Question 5 and the central values do not seem to be very reliable either. However, it is worth noting that the upper years of the 2021/2022 cohort and the two earlier cohorts scored higher than the lower years. Likewise, according to the study by Arruti and Paños-Castro (2023), just over 32% of the participating teachers claimed to clearly distinguish between the social, cultural and economic values that can be generated through entrepreneurial projects.

In the case of Question 6, the highest scores were at level 3 in all years and cohorts, i.e., they believed that the EC should be taught in PE, an aspect that has been advocated by the Comisión Europea (2006) for years. This is why it would be advisable to follow the recommendations of Lackéus (2015) and Seikkula-Leino et al. (2021), who considered that students can be motivated by proposing actions through which they can create value for other people, and that ‘infusing value creation experiences across the entire curriculum can be one of the most important contributions entrepreneurship can make to education in the future’ (Lackéus, 2015, p. 16). In any case, according to Arruti and Paños-Castro (2023), 30% of the participating teachers saw themselves as capable of encouraging their students to generate innovative ideas or projects that add value, based on real and changing challenges and needs of their environment, and highlighted the importance of managing uncertainty, ambiguity and risk. The problem is that they did not put this into practice, as confirmed by Seikkula-Leino et al. (2021), or if they did, it was not based on the real challenges and needs of their environment.

In relation to Question 7, H4 can be accepted. It should be noted that 50% of the participants chose the highest option (level 4), which was also the most favored option for all academic years and cohorts. All of them considered that EE involves taking the needs, interests and talents of pupils as a starting point in order to guide them, and working with them so that they can autonomously acquire the ECs. These data are in line with the results achieved by Arruti and Paños-Castro (2023), also in accordance with Azqueta and Naval (2019).

As far as Question 8 is concerned, the results lead to rejecting H5. It seems that belonging to one or another cohort/academic year is related to the self-perceived level of knowledge of Assessment in relation to the need to assess and give feedback about the EC. Moreover, as in Questions 5 and 7, the dispersion of data was very high and the central values did not seem to be very reliable. It is striking that more than 10% of respondents did not consider assessing the EC, compared to 31% who believed that it is important to explicitly assess the ECs, but did not feel able to do so.

In turn, the results gathered in Question 9 lead to H5 being accepted. It is worth highlighting that approximately 50% of participants (all academic years and cohorts) selected level 4 as their preferred response option, and advocated helping pupils during the assessment process so that they can self-assess, manage their learning, effort, achievements and progress, and reflect critically on their entrepreneurial learning process. These data are in line with the recommendations made by Lackéus (2015), Penaluna and Penaluna (2015), and Pérez García (2021), who argued for the role of learners and their ability for self-reflection on the entrepreneurial learning process:

through self-reflection that has led to self-direction, the student has new problems to consider and new opportunities to look far and wide for solutions, at which point the cycle reverts to convergent thinking that eliminates poor ideas and offers new insights (Penaluna and Penaluna, 2015, p. 24).

Finally, in relation to Question 10, the results obtained show that H6 must be accepted. The vast majority of respondents chose between the second response option (I think it is important to provide opportunities for my students to share and implement entrepreneurial ideas and actions with different groups of people) and the fourth option (I encourage/will encourage my students to identify possible groups of people with whom to share and implement entrepreneurial ideas and actions). These data are in line with research by Lackéus (2015) and Deveci and Seikkula-Leino (2018).

However, only 13% of the teachers participating in a study by Arruti and Paños-Castro (2023) claimed to support their students in the management of internal and external actors with whom they share achievements and progress. This contrasts with Miço and Cungu (2023, p. 9), who corroborated that 45.5% of the participants in their study ‘try to create connections and cooperation structures with businesses and community organizations to support the entrepreneurship curriculum in their schools’. It could be why, Sanz Ponce and Núñez Canal (2022, p. 143) proposed ‘designing achievement tests that actively include the recipients or customers of the products or services made by the students in the entrepreneurial projects, specifically devised for each level of education and educational practice.’

## 6 Conclusion

The aim of this study was to analyze the level of EC knowledge of prospective teachers who are taking or have recently taken the DPEUD. The analysis was based on two sub-competences of the EC, namely, entrepreneurial knowledge and understanding, and assessment, which, according to Arruti et al. (2021), are not included in the curricula of the degree in PE at the University of Deusto, Spain. Specifically, the aim was to examine the level of knowledge of both sub-competences as perceived by the participating pre-service teachers. This was done by focusing on the definition of EE, the European EC benchmark frameworks Entrecomp and EntreCompEdu, and the social, cultural and economic value of entrepreneurial actions in schools. The analysis also included that the needs, interests and talents of students should be included in the development of the EC, the need to assess and give feedback on them, and the need to identify opportunities and groups to share and implement entrepreneurial ideas and actions.

With regard to entrepreneurial knowledge and understanding, in the light of the results it is necessary to further emphasize that EE should be part of the different national teacher training strategies, and not only of student education, as is the case in Spain pursuant to Royal Decree 157/2022 (Ministerio de Educación y Formación Profesional, 2022). This education will help teachers to feel prepared and more confident about their initial training to provide their pupils with skills and knowledge to implement entrepreneurial projects and actions in the near future. In line with Seikkula-Leino et al. (2021), awareness of the EntreComp and EntreCompedu frameworks, which are still known to only a very small number of students, and how to implement them could be an appropriate way to start this process.

Furthermore, we believe it is advisable for policy makers to consider and discuss EE in the different educational frameworks, laws and decrees, so that a consensus can be reached between all the relevant stakeholders on how to conceive EE and entrepreneurship, and on how to plan and assess entrepreneurial actions. This could help to break down the inconsistencies between entrepreneurial goals and practices (Seikkula-Leino et al., 2010; Penaluna and Penaluna, 2015).

The results of this study also lead to the conclusion that it is not enough to know about the EC and its importance in PE (Comisión Europea, 2006); it is also necessary to have an impact on the students’ ability to create social, cultural or economic value through entrepreneurial actions (Lackéus, 2015).

In relation to Assessment, emphasis should be placed on the need to assess the EC and, above all, on the knowledge and use of various techniques and strategies to facilitate this process. There is still also a need to focus on self-assessment by pupils, so that they can manage their learning, as they should critically reflect on their entrepreneurial learning process (Lackéus, 2015; Penaluna and Penaluna, 2015; Pérez García, 2021). To this end, it is important for teachers to take a stand and motivate their pupils to identify possible groups of people with whom to share and implement entrepreneurial ideas and actions.

The limitations of the study include the fact that it used a non-probabilistic convenience sample and therefore it is only possible to make descriptive statements about the sample. For this reason, it would be interesting for future research to increase the size of the sample by using simple random sampling of all future students of the Bachelor’s Degree in Primary Education in Spain. Apart from that, as future research we propose to deepen on the following topics: the way entrepreneurial competence is assessed in the different national curricula at a macro-, meso- and micro- level (teaching-learning process); the way European entrepreneurial frameworks and other similar ones are implemented in the different pre-service and in-service teachers training programs; and, the different key elements that an entrepreneurial teacher training program must consider.

## Data availability statement

The raw data supporting the conclusions of this article will be made available by the authors, without undue reservation.

## Author contributions

AA: Conceptualization, Methodology, Project administration, Supervision, Visualization, Writing – original draft, Writing – review & editing. EB: Conceptualization, Formal analysis, Methodology, Visualization, Writing – review & editing. JP-C: Conceptualization, Methodology, Visualization, Writing – review & editing.
